# The Subnational Human Development Database

**DOI:** 10.1038/sdata.2019.38

**Published:** 2019-03-12

**Authors:** Jeroen Smits, Iñaki Permanyer

**Affiliations:** 1 Global Data Lab, Institute for management Research, Radboud University, Nijmegen, Netherlands; 2 Centre d’Estudis Demogràfics, Universitat Autònoma de Barcelona, Bellaterra, Spain

**Keywords:** Public health, Education, Economics, Developing world

## Abstract

In this paper we describe the Subnational Human Development Database. This database contains for the period 1990–2017 for 1625 regions within 161 countries the national and subnational values of the Subnational Human Development Index (SHDI), for the three dimension indices on the basis of which the SHDI is constructed – education, health and standard of living --, and for the four indicators needed to create the dimension indices -- expected years of schooling, mean years of schooling, life expectancy and gross national income per capita. The subnational values of the four indicators were computed using data from statistical offices and from the Area Database of the Global Data Lab, which contains indicators aggregated from household surveys and census datasets. Values for missing years were estimated by interpolation and extrapolation from real data. By normalizing the population-weighted averages of the indicators to their national levels in the UNDP-HDI database, values of the SHDI and its dimension indices were obtained that at national level equal their official versions of the UNDP.

## Background & Summary

The Human Development Index (HDI) published yearly since 1990 by the United Nations Development Program (UNDP) is perhaps the most popular index used to assess countries’ well-being levels across the globe. Defined as an average of achievements in health, education and standard of living, its popularity can be attributed to the simplicity of its characterization and to its underlying message that “development is more than economic growth”^1^.

A disadvantage of the HDI is that it is a national level aggregate that could potentially hide many disparities within countries. Since individuals and regions within countries tend to differ in educational attainment, health status and standard of living, national averages like the HDI inevitably ignore existing differences. Indeed, existing disparities in health, education and asset ownership have motivated the United Nations to include the ‘reduction of inequalities between and within countries’ as Goal#10 of the 17 Sustainable Development Goals (SDGs), which form the global development agenda in the coming decades. The Subnational Human Development Index (SHDI) Database (Data Citation 1) presented here offers for 1625 regions in 161 countries for the period 1990-2017 the subnational HDI and indicator data needed for monitoring progress with regard to key aspects of this agenda.

There have been some earlier attempts to uncover within-country variation in human development along different lines, like income groups^2,3^, migrants and nonmigrants^4^, and municipalities^5,6^. However, these efforts only produced subnational HDI data for a handful of countries. More recently, Kummu *et al.*^7^ presented a gridded global dataset for GDP and HDI, including HDI data at 5 arc-minute resolution for 1990–2015. This is a major step forward compared to the earlier efforts. However, the value of this gridded dataset for studying subnational human development remains restricted, as the HDI part is based on subnational data for only 39 countries, the majority of which belonging to the high human development group. Subnational data on low and middle-income countries (LMICs) – which are most important for the global development agenda – are to a large extent lacking.

Our SHDI Database improves on the Kummu *et al.*^7^ database in important ways. First, it contains subnational data for 161 countries, covering all regions and development levels of the world. Second, besides subnational data on the HDI, it also provides subnational data on the indicators and dimension indices used for constructing the HDI, which are important development indicators themselves. Third, all countries included in the SHDI database have real subnational data for at least two of the three dimension indices, and most of them (90%) have subnational information for all three indices. Existing data gaps (missing years) could in most cases be filled in with interpolation or extrapolation over a short (1–5 years) time period. Only in 35% of cases extrapolation over a longer time period had to be used. Our most important contribution is with regard to LMICs. For most of these countries, until recently hardly any subnational data was available. However, since 2016 the Area Database of the Global Data Lab (https://www.globaldatalab.org) provides a large number of development indicators at subnational level for over 100 LMICs. Much of the data for LMICs used to construct the SHDI database was obtained from this GDL Area Database.

The indicators in the SHDI Database are scaled in such a way that their population weighted averages equal their national values in the official UNDP-HDI database. This procedure, which was also used by Kummu *et al.*^7^, guarantees that at the national level, the indicators, dimension indices and SHDI values are equal to the values used by the UNDP.

The SHDI database provides researchers worldwide with high-detail contextual variables that improve our understanding in wide-ranging areas of the social and behavioural sciences (including family formation, migration, health and mortality, epidemiology, cultural/ideational/normative change, religion, socio-economic change, or environmental sustainability). In the policy-making arena, the subnational SHDI is very pertinent for the global development agenda, as it can help directing resources to the places they are mostly needed. By moving beyond country-level averages, the SHDI has the potential to bring equity concerns to the fore, and to serve as a tool to articulate national and international development policies into a coherent whole.

## Methods

The Subnational Human Development Index (SHDI) Database (Data Citation 1) discussed in this paper presents a translation of the UNDP’s official HDI (http://hdr.undp.org) to the subnational level. Similar to the HDI, the SHDI is an average of the subnational values of three dimensions: education, health and standard of living. In its official version defined at the national level, the indices measuring these dimensions are constructed on the basis of four indicators. Table 1 shows some characteristics of these indicators. For the health dimension *life expectancy at birth* (LEXP) is used as indicator. For standard of living, (the log of) *gross national income per capita* (LGNIc) is used *–* measured with Purchasing Power Parities (PPP) in 2011 US$. The educational dimension is measured with two indicators. The first one, *mean years of schooling of adults aged 25+ *(MYS), reflects the current situation with regard to education in a society. The second one, *expected years of schooling* (EYS), indicates the future level of education of the population. EYS is defined as the number of years of schooling a child of school entrance age can expect to receive, if prevailing patterns of age-specific enrolment rates persist throughout the child’s schooling life. When computing the dimension index for education, the values of MYS and EYS are weighted equally.

The SHDI Database includes SHDI, dimension indices and indicators for 1625 regions. The number of subnational regions varies between countries, from two regions for very small countries (Comoros, Malta) to 51 for the USA, with an average of 10. The years for which the data are available are similar to those of the UNDP HDI. For most countries, SHDI values are presented for the period 1990–2017, but for some countries the time period is shorter. The total number of country-years for which SHDI data is available is 4087.

In the following sections, we first discuss in detail the major data sources used for creating the SHDI database. After that, we discuss for each of the three dimensions separately how the indicators for the dimensions were measured. Thereafter, in the “Data Processing” subsection, two measurement issues are discussed. First, we report how the subnational values of the indicators derived from statistical offices and the Global Data Lab were connected with their national values obtained from the HDI database of the UNDP. Second, we discuss the techniques used to fill in data gaps, in order to obtain yearly values for the period 1990–2017. Finally, we explain how the different dimension indices are computed and how these are combined to generate the SHDI.

### Data sources

Three major data sources were used to create our SHDI database. We approached statistical offices, including Eurostat, the statistical office of the European Union (https://ec.europa.eu/eurostat), by email communication or visiting their websites to obtain data. We downloaded data from the Area Database of the Global Data Lab (https://www.globaldatalab.org). And we downloaded data from the HDI website of the Human Development Report Office of the United Nations Development Program (http://hdr.undp.org). In the ‘SHDI Start’ data file (Data Citation 1), for each country information is provided on the data source(s) used for the subnational values of the indicators. In this file also for each country the years for which data is available, the number of subnational regions and the population size is presented. Below we discuss the three main data sources in more detail.

#### Statistical offices

For most EU countries the data was derived from the Eurostat database (https://ec.europa.eu/eurostat/data/database). The definition of subnational areas used by Eurostat is based on the NUTS classification (Nomenclature of territorial units for statistics, https://ec.europa.eu/eurostat/web/nuts), a hierarchical system for dividing up the economic territory of the EU. NUTS1 are the major socio-economic regions and NUTS2 basic regions for the application of regional policies. For most EU countries, data was used at NUTS2 level. For Germany and the UK this level is so detailed that data at the NUTS1 level was used. For some EU countries, no subnational data could be obtained from Eurostat and other sources had to be used. For Estonia, Ireland, Lithuania, Latvia, Malta and Slovenia, data from their national statistical offices was used. For Cyprus and Luxemburg no subnational data could be obtained.

Eurostat data for mean years of schooling was available for 2000–2017, for expected years of schooling from 2013–2016, for GDP in Euros PPP from 2004–2016, and for life expectancy at birth from 1990–2016. For Australia, Canada, China, Croatia, Japan, New Zealand, South Korea, Russia, and the USA, data from national statistical offices was used. For South Korea and Russia, no usable educational data could be derived from their statistical offices. For these countries, data on education was derived from survey datasets. For Russia, data from the European Social Survey for 2012 and 2017 were used. For South Korea, data from the World Values Survey 2010 was used.

#### GDL Area Database

The Global Data Lab provides since 2016 freely downloadable subnational development indicators for LMICs through its Area Database (GDL-AD; https://www.globaldatalab.org/areadata). These indicators are constructed by aggregation from representative survey and census datasets. The major data sources used by GDL for this purpose are Demographic and Health Surveys (DHS, https://www.dhsprogram.com), UNICEF Multiple Indicator Cluster Surveys (MICS, http://mics.unicef.org) and datasets from population censuses distributed by IPUMS International (https://international.ipums.org). These sources provide large samples, often 50,000 to 100,000 or more respondents, containing information on all household members. For LMICs for which these sources are not available, GDL uses other – country-specific – surveys, or less comprehensive data sources, like Afrobarometer or Americas barometer surveys (http://www.afrobarometer.org, http://www.americasbarometer.org), which include only adults instead of complete households.

For most LMICs, GDL-AD provides the two indicators needed for creating the educational index, mean years of schooling and expected years of schooling. However, the indicators needed for the health and income dimensions are usually not available in the required form in household survey and census datasets. The subnational values of these indicators for LMICs are therefore estimated using data on child mortality and household wealth that is derived from GDL-AD.

#### UNDP Database

The third database used for constructing the SHDI database is the database with national development indicators maintained by the Human development Report Office of the United Nations Development Program (http://hdr.undp.org/en/data). This database contains time series for the period 1990–2017 for the HDI, its dimension indices, and the indicators used for creating the dimension indices, plus a large number of other socio-economic, health, education, demographic and environmental indicators. From this database, the national data is derived that is used to scale the SHDI indicators to their UNDP values.

For Kosovo, Somalia and Taiwan, no national data were available in the UNDP database. For Kosovo, data for 2015 was derived from the national Human Development Report 2015^8^. For Somalia national GDP per capita was derived from the World Bank’s World Development Indicators (http://wdi.worldbank.org) and schooling data for 2012 from the national Human Development Report 2012^9^ and from GDL-AD for 2006. For Taiwan, data from the Taiwanese Directorate General of Budget, Accounting and Statistics was used (http://eng.stat.gov.tw/ct.asp?xItem=25280&ctNode=6032&mp=5). Taiwan and Hong Kong are included in the SHDI Database among the provinces of China.

### Estimating the SHDI components

#### Education dimension

For the educational dimension, data on mean years of schooling of the adult (25+) population and data on expected years of schooling of children aged 6 are required. Mean years of schooling indicates the current schooling level of the population. Expected years of schooling indicates the future schooling level.

For most LMICs, both variables could be directly obtained from GDL-AD. Mean years of schooling was computed by GDL-AD taking for each region the average number of years of education completed by adults aged 25 and over in the survey and census datasets. In most of these datasets educational level is measured in years of education completed, so that mean years of schooling could be computed rather straightforwardly. In a restricted number of cases, education was measured by the highest completed education level. In those cases, the data was turned into years of education on the basis of information on the number of years it normally takes to complete a certain level in the specific country (often six years for primary, 9 years for lower/junior secondary, 12 years for upper/senior secondary, fifteen years for a bachelor degree and sixteen or seventeen years for a master degree.

To compute expected years of schooling for LMICs, data on educational attendance for children aged 6 to 24 in the regions was used. For each year-group (6, 7, 8…24), the share of children attending school was determined and these shares were added up. The sum of these percentages represents the number of years of schooling a child of school entrance age (age 6) can expect to receive, if prevailing patterns of age-specific enrolment rates of children aged 6–24 would persist throughout the child’s schooling life. For LMICs for which only samples of adults were available, expected years of schooling could not be computed and only mean years of education was available. For these countries, we estimated the subnational values of expected years of schooling by applying the variation in mean years of schooling to the national value of expected years of schooling obtained from the UNDP database, using the following formula:

(1)Ei=EnMiMn

whereby *M*_*i*_ and *E*_*i*_ are the mean and expected years of schooling of region *i* and *M*_*n*_ and *E*_*n*_ the national values of mean and expected years of schooling in the UNDP database.

For HICs, the data on schooling is generally derived from statistical offices. This means that for part of these countries, data on expected years of schooling was lacking, as this data is often not available at statistical offices. An exception is Eurostat, which provides for many EU countries in recent years subnational data on the number of children enrolled in school by age and the total number of children by age, so that for each age in the 6–24 age group the percentage of children in school can be computed. For most EU countries we therefore could include expected years of schooling.

Data on mean years of schooling derived from statistical offices and Eurostat is generally available in the form of tables with the numbers or percentages of children at the different educational levels. This data was turned into years of education on the basis of information on the number of years it normally takes to complete a certain level (as discussed above).

For 23 countries (Australia, Chili, Cape Verde, Ecuador, Ireland, Canada, China, Cuba, Estonia, Croatia, Japan, South Korea, Kuwait, Lebanon, Libya, Lithuania, Malta, Mauritius, New Zealand, Russia, Saudi Arabia, Slovenia, USA), no data on expected years of schooling was available. For these countries the subnational variation in mean years of schooling was applied to the national UNDP value of expected years of schooling as discussed above (using Equation 1). For Latvia, neither expected years of schooling nor mean years of schooling was available. For this country, the national UNDP values for these indicators were used for the subnational regions.

**Figure 1:**
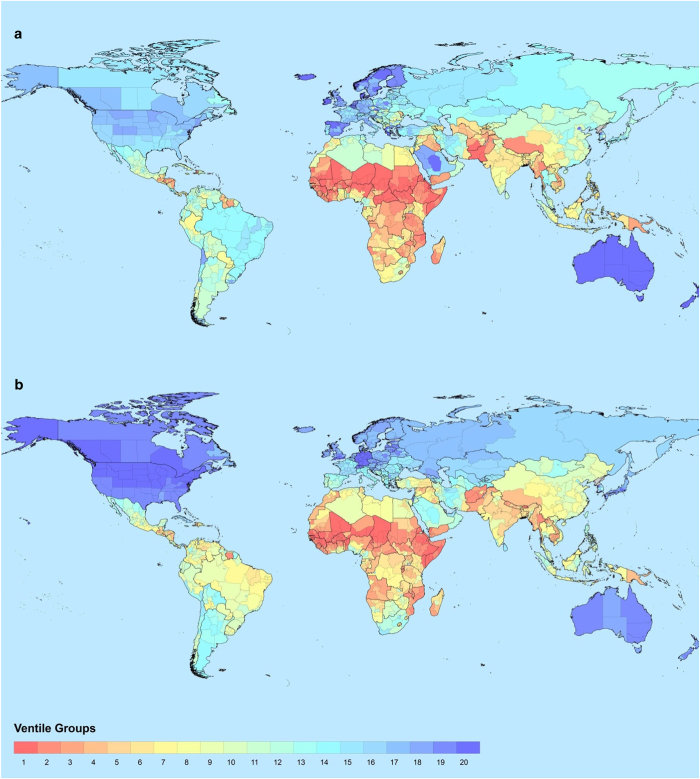
World maps with the distribution of the education dimension indicators. (**a**) Distribution of EYS values. (**b**) Distribution of MYS values.

Figure 1a,b show the subnational variation in expected years of schooling and mean years of schooling across the globe in 2017.

#### Standard of living dimension

For the economic dimension of the HDI, the natural logarithm of Gross National Income per capita in 2011 US$ PPP (LGNIc) is used as indicator. For HICs and some middle-income countries (MICs), subnational values of LGNIc were based on data derived from national statistical offices and Eurostat. Data often deviated from the required definition in that GDPc instead of GNIc was available, that local currencies instead of 2011 US$ was used, and/or that no adjustment for PPPs was applied. These issues were not very problematic, as the data was normalized on the basis of national LGNIc values with the correct definition derived from UNDP.

For most LMICs, data on standard of living were derived from the GDL-AD. Given that household surveys and censuses for LMICs often do not contain information on income and, if they do, this information is not very reliable in poor areas, subnational values of LGNIc were estimated based on household wealth. For this purpose, the International Wealth Index (IWI) was used, which measures household wealth on the basis of information on asset ownership, housing quality and access to public services^10^. The IWI scale runs from 0 to 100, with 0 meaning ownership of none of the assets and bad quality housing and services and 100 indicating ownership of all assets and best quality housing and services.

To estimate LGNIc for the subnational regions on the basis of IWI, a regression model was constructed that explained the variation in national LGNIc derived from the UNDP database on the basis of national IWI scores derived from GDL-AD. We compared models with linear and nonlinear effects of IWI on the basis of their adjusted R^2^ and found the linear model to have the best fit. Besides IWI, the prediction model included controls for year and global regions. This model had an explained variance of 82.6%, which is considered a good fit for this kind of data^11^. The global regions that were distinguished are Central America and the Caribbean, South America, West Africa, Central Africa, Southern Africa, East Africa, Middle East and North Africa, Central Asia, South Asia, East and South-East Asia and Pacific, Eastern Europe. An additional indicator was included to address the relatively high GNIc of the following oil-exporting countries: East Timor, Equatorial Guinea, Gabon, Kazakhstan, Kuwait, Saudi Arabia and Turkmenistan.

On the basis of this prediction model, the subnational values of LGNIc were estimated, which were further improved by scaling them on the basis of national LGNIc values derived from the UNDP database. For Cuba, LGNIc could not be estimated as no information on household wealth was available. For this country the national UNDP value was used for the subnational regions.

**Figure 2:**
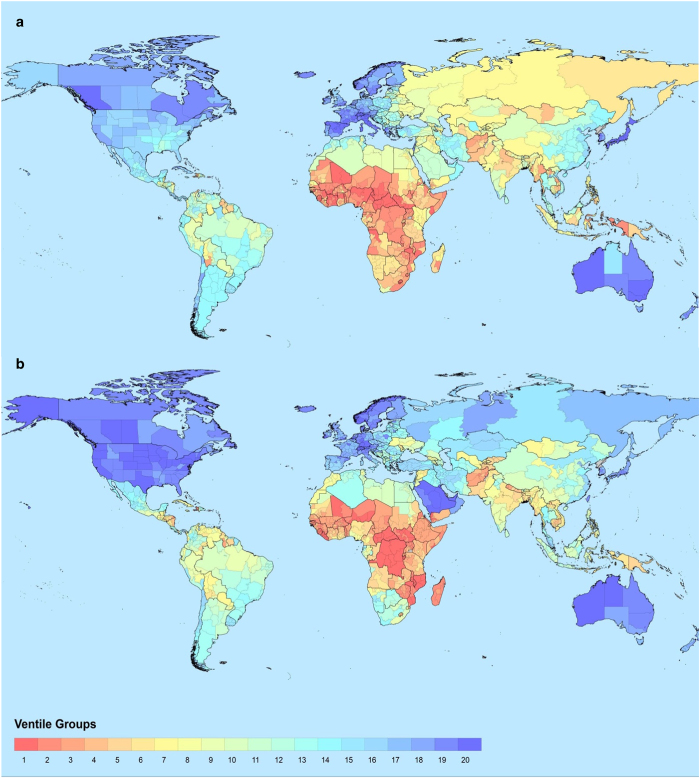
World maps with the distribution of the Standard of Living and Health dimension indicators. (**a**) Distribution of LGNIc values. (**b**) Distribution of LIFEX values.

Figure 2a shows the subnational variation in LGNIc across the globe in 2017.

#### Health dimension

The health dimension of the HDI uses life expectancy at birth (LEXP) as indicator. For HICs and some MICs, subnational values of LEXP were based on data derived from national statistical offices and Eurostat. For LMICs, data were derived from the GDL-AD. Given that household surveys and censuses generally do not contain information on LEXP, subnational values of this indicator were for these countries estimated based on information on under-5 mortality (U5M).

To estimate LEXP on the basis of U5M, a regression model was constructed that explained the variation in national LEXP derived the UNDP database on the basis of national U5M scores derived from GDL-AD. We compared models with linear and nonlinear effects of U5M on the basis of their adjusted R^2^ and found the nonlinear model including both U5M and U5M^2^ to have the best fit. Besides U5M and U5M^2^, the prediction model included controls for year, global region (see above) and an additional indicator for the exceptional low life expectancy in Rwanda in the early 1990s. The selected model had at the national level an explained variance of 89.1%, which is considered a good fit for this kind of data^11^. The subnational values of LEXP estimated on the basis of this model were further improved by scaling them on the basis of the national LEXP values derived from the UNDP database.

For 16 countries (Argentina, Bosnia and Herzegovina, Barbados, Cape Verde, Costa Rica, Ecuador, Croatia, Kuwait, Lebanon, Libya, Malta, Mauritius, Malaysia, Panama, Saudi Arabia, Uruguay) no data on life expectancy or U5M was available. For these countries, the national UNDP values of LEXP were used for the subnational regions.

Figure 2b shows the subnational variation in life expectancy across the globe in 2017.

### Data Processing

#### Scaling the indicators

To obtain the best possible estimates for the four indicators given the data limitations, we have taken their national values from the UNDP-HDI database and scaled the subnational values in such a way that their population weighted mean for a given year equals the national UNDP value for that year. In this way, we obtained indicators that on the one hand display as well as possible the subnational variation of the data available at statistical offices and in survey datasets, while on the other hand their population weighted national averages are equal to the values used by the UNDP in constructing the HDI.

**Figure 3:**
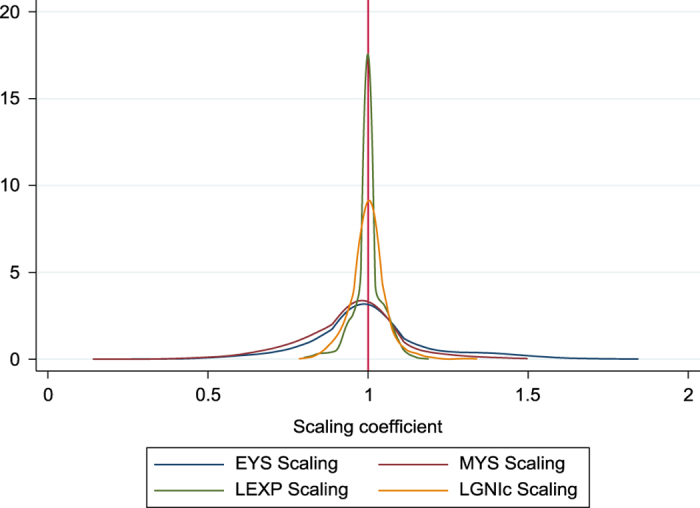
Density functions of the scaling coefficients corresponding to EYS, MYS, LEXP and LGNIc.

For each country-year-SHDI indicator combination, we have a multiplicative scaling coefficient that inflates/deflates the subnational estimates in such a way that their population-weighted averages coincide with the corresponding UNDP value. By definition, these scaling coefficients take the value of one when no re-scaling is necessary. Figure 3 summarizes the extent of scaling we have performed on the indicators, by showing the density plots corresponding to the distribution of scaling coefficients for EYS, MYS, LEXP and LGNIc separately. As can be seen, the density plots are roughly symmetric, concentrated around the value of one. That is: the amount of scaling is relatively small (i.e. near the ‘no-scaling’ value of one) and goes in either direction roughly the same number of times (i.e. we had to ‘inflate’ as often as ‘deflate’ the estimates). The scaling coefficients vary more widely for the education variables than for LEXP and LGNIc. For the last two indicators, the extent of re-scaling is remarkably small, as the range of values of the scaling coefficients is very narrow.

#### Addressing missing years

Given that household surveys and censuses are not held every year, for many countries the indicators are only available for a restricted number of years. To obtain their values for the whole period 1990–2017, the missing information was estimated by interpolation or extrapolation techniques. This estimation process was facilitated by the fact that the UNDP Database contains the national values for all four indicators for each year in this period, which means that only the subnational variation had to be interpolated or extrapolated.

If information on the indicator value for both a preceding and a succeeding year was available, the values of the indicator for the subnational regions in the missing year were initially estimated by linear interpolation from the region’s value in the earlier end later year. The obtained values were subsequently rescaled so that their population-weighted averages were equal to the national values of the indicator derived from the UNDP database. In this way indicators were obtained that over time follow exactly the variation of the respective national indicators in the UNDP database, while at the same time their subnational variation is in between the subnational variation in the earlier end later year.

If the subnational indicator values were only available for an earlier or a later year, extrapolation had to be used. This was also done in two steps. First, the values of the indicator for each subnational region in the missing year were filled in by taking the region’s indicator in the nearest year for which real information was available. Second, the obtained values for the regions were rescaled so that their population-weighted averages were equal to the national values of the indicator derived from the UNDP database. The indicators constructed in this way follow over time exactly the variation of the respective national indicators in the UNDP database, while their subnational variation follows the pattern of subnational variation in the nearest year with real information. This approach is similar to the procedure used by Kummu *et al.*^7^.

Of the total number of 4087 country-year combinations, 28.2% was based on real data for at least one indicator, 24.1% was interpolated, 25.9% was extrapolated over a short time period (0–5 years) and 21.8% was extrapolated over a longer time period. After 2000, the situation is more favorable, with only 10.5% of country-year combinations estimated over a longer time period. Information on the size of the errors due to interpolation and extrapolation is provided in the Technical Validation section.

### Dimension indices and SHDI

To create the dimension indices and SHDI on the basis of the four indicators discussed above the same procedures were used as are used by the UNDP to compute the regular HDI^12^. For computing the dimension indices, the following formula is used:

(2)Dimensionindex=actualvalue−minvaluemaxvalue−minvalue

The minimum and maximum values are the so-called ‘goalposts’, which are used to take care that the values of the dimension indices remain between 0 and 1 (see Table 1). For life expectancy at birth the UNDP goalposts are 20 and 85. For standard of living they are 100 and 75,000. For expected years of schooling they are 0 and 18, and for mean years of schooling 0 and 15. To obtain the dimension index for education, the geometric mean of the separate indices for expected years of schooling and mean years of schooling is taken.

To compute the SHDI on the basis of the three dimension indices, the geometric mean of the three indices is taken:

(3)SHDI=(Education⋅Health⋅Income)1/3

For a few regions, the value of one of the education indicators was higher than the maximum goalpost. In these cases, the values were capped at the goalpost levels.

### Code availability

The database was constructed with SPSS-25. The starting dataset with all input data combined and the data processing code file for inter/extrapolation and for index construction are available at Data Citation 1.

## Data Records

The dataset of the Subnational Human Development Index (SHDI) Database (Data Citation 1) contains four data files: “SHDI-Database”, “SHDI-Starting-Data”, “SHDI-Data-Quality” and “UNDP-HDI-Data”. All files are available as SPSS, STATA and Excel files.

The *SHDI-Database* file contains the subnational and national values of the four indicators, the three dimension indices and the SHDI for each year from 1990 to 2017 for 1625 subnational regions in 161 countries, which together cover over 99% of the world population. National values in this file are equal to the corresponding values in the UNDP HDI database. The *SHDI-Starting-Data* file contains the data as obtained from the original sources, presented in a standard form, so that it can be analysed in a straightforward way. This file also contains information on the data sources used for each country-year combination, the number of subnational regions used for each country and the population size of the regions. The *SHDI-Data-Quality* file contains for all possible country-year combinations and for all four indicators information on whether the subnational values of the indicator were actually available in a given year, or whether they were interpolated or extrapolated, and, if so, over how many years. This information is extremely useful for researchers who want to have an overall picture of the data quality for specific time periods and/or want to restrict their analysis to country-year observations satisfying certain data quality requirements. The *UNDP-HDI-Data* file contains the national values of the HDI and the four indicators and the national population size for all country-year combinations. The descriptions of the records of the four data files are presented in Tables 2, 3, 4, 5. The SHDI data is also available in a more easy to use form on https://hdi.globaldatalab.org.

## Technical Validation

The major analytical steps in the process of creating the Subnational Human Development Database were the construction of the three dimension indices and subsequently the SHDI on the basis of the four indicators obtained from national statistical offices, Eurostat and GDL-AD. The degree of precision of the subnational estimates depends on the quality of the data sources used in the construction of the indicators. Since Eurostat and the statistical offices of HIC are well organized and funded organizations, the data they generate satisfy the highest quality standards. The survey and census datasets used by GDL to create GDL-AD are coordinated by renowned and experienced organizations like the DHS Program, UNICEF, and IPUMS International, and are the same data sources that are routinely employed by national and international institutions to document the socio-demographic characteristics of LMICs. Hence, the SHDI database is based on the most reliable sources of socio-demographic and economic data one can currently work with.

**Figure 4:**
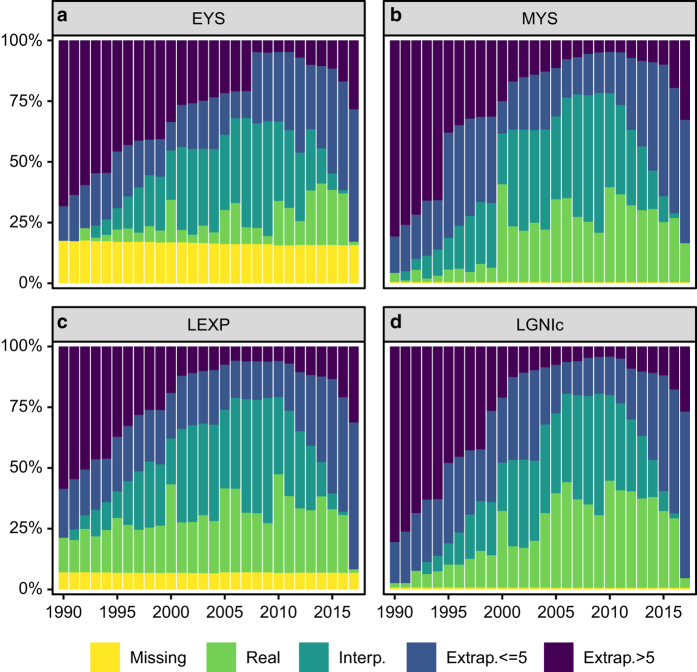
Data processing statistics of the SHDI indicators over time. The different colours indicate the share of ‘Missing’, ‘Real’, ‘Interpolated’, ‘Extrapolated by 5 or less years’ and ‘Extrapolated by more than 5 years’ observations for EYS (**a**), MYS (**b**), LEXP (**c**), LGNIc (**d**) from 1990 up to 2017.

Besides the sources of the data, there are three other potential causes of quality loss: (1) the lack of subnational data for one or more indicators, (2) the procedures used to estimate LGNIc and LEXP on the basis of data on IWI and U5M, and (3) the procedures used to estimate subnational indicator values for the years these values were not available. Regarding the first point, Fig 4a–d show the percentage of country-year observations in which the different SHDI indicators were either (1) observed, (2) interpolated, (3) extrapolated by 5 years or less, (4) extrapolated by more than 5 years, or (5) missing, from 1990 to 2017. As can be seen, the patterns are similar for the four indicators used in the construction of the SHDI. The share of highest quality observations (i.e. real or interpolated values) is relatively small in the 1990s, but includes over half of the observations for almost the complete period 2000–2017. Only in the last year, there is a decrease, due to the fact that not all subnational 2017 data has become available. MYS and LGNIc have the highest percentage of true observations and EYS the least. The share of real and interpolated estimates follows the same pattern for the four SHDI indicators: it increases over time to reach a maximum, and decreases when approaching the upper limit of 2017 (as interpolations are not possible at the extremes of the observation window). In 2009 (the year for which the data is best), the share of real and interpolated values varies between 50.5 and 79.5% (the shares for EYS and LGNIc, respectively).

Regarding extrapolation, we distinguish between ‘short-term’ and ‘long-term’ versions depending on whether the number of extrapolation years is up to or above five. In general, for the four SHDI indicators the share of short-term extrapolations is roughly stable up to 2005 (accounting for around 15–30% of the observations), then decreases somewhat between 2005 and 2010 (i.e. the years when real and interpolated data are highest) and increases again between 2010 and 2017. The trends in the shares of long-term extrapolations are very similar across indicators. They are rather large for the initial years of the observation window and decrease over time to reach a minimum around 2010 (where they only account for around 4% of the observations) and then increase slightly until 2017.

Table 6 presents information on the joint distribution of the four indicators. To simplify the presentation, the quality of the estimates is classified in two broad categories: ‘High-quality data’ (i.e. real or interpolated data), and ‘Lower-quality data’ (i.e. short- and long-term extrapolations). As shown in Table 6, in 52.3% of the country-year combinations there is at least one of the three SHDI dimensions that is calculated with a high-quality indicator. Analogously, 44.7% and 34.5% of the SHDI country-year combinations are based on at least two and three high-quality indicators, respectively. These numbers improve considerably over time. Considering the post-2000 period only, 61.9, 58.5 and 45.4% of the country-year SHDI combinations are based on at least one, two and three high-quality indicators, respectively.

**Figure 5:**
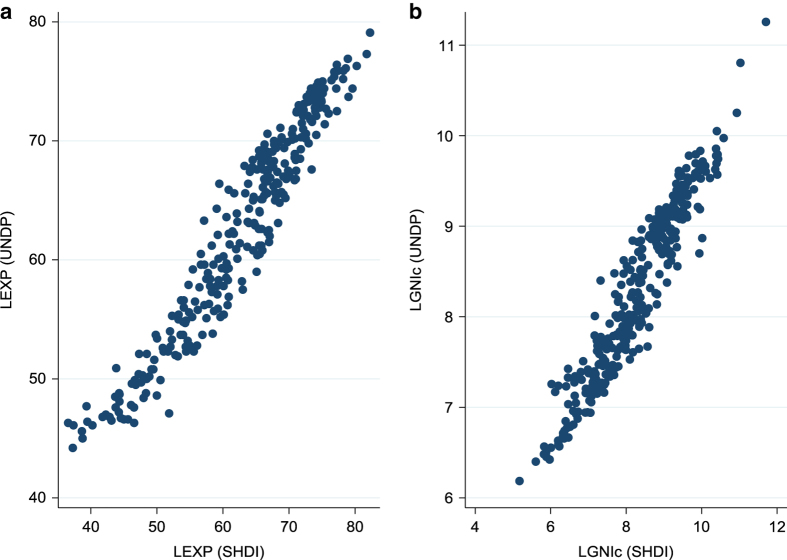
Comparison between real and model-based national-level estimates for the Health and Standard of Living dimension indicators. (**a**) Comparison for Life expectancy at birth. (**b**) Comparison for Log GNI per capita.

As regards the second potential source of quality loss (because of the need to estimate LGNIc and LEXP for most LMICs), we have to rely on indirect measures to assess the quality of our estimates, like the explained variance of the models and the quality of their national-level predictions. With an explained variance of 82.6 and 89.1% respectively, the models fit substantially above the value of 50 percent which according Studenmund^11^ can be considered a good fit for this kind of cross-sectional data. Regarding the quality of the predictions, we present in Fig. 5 the differences between the national predictions on the basis of the models and the known national values. Although there is some variation, overall the predictions are very close to the real national values. We therefore assume that also at the subnational level the models will provide good predictions of the values of these indicators.

Regarding the third point (quality loss due to the estimations to fill in data gaps), we expect the size of the loss to depend on the estimation procedure that could be used (interpolation or extrapolation) and the number of years over which estimation took place. To estimate the error that might originate from the inter- and extrapolations, we have performed an error analysis for each SHDI indicator separately (i.e. for EYS, MYS, LEXP and LGNIc), using the following approach derived from Kummu *et al.*^7^ Taking the set of real observations as starting point, we generated a set of *simulated* interpolated and extrapolated values which we subsequently compared with the observed ones to quantify the degree of agreement and the approximate error size.

To illustrate: if a certain indicator is observed in years *t*_*1*_, *t*_*2*_ and *t*_*3*_ (with *t*_*1*_<*t*_*2*_<*t*_*3*_), we have generated (i) the forward extrapolations of *t*_*2*_ and *t*_*3*_ from *t*_*1*_, (ii) the forward extrapolation of *t*_*3*_ from *t*_*2*_, (iii) the backward extrapolations of *t*_*1*_ and *t*_*2*_ from *t*_*3*_, (iv) the backward extrapolation of *t*_*1*_ from *t*_*2*_, and (v) the interpolation of *t*_*2*_ from the values in *t*_*1*_ and *t*_*3*_. In all those cases, the simulated value is compared with the observed one via the formula 100·|*sim*−*obs*|/*obs*, which we denote as ‘relative error’. Running over all possible such combinations that the set of observed data allows, we have generated a distribution of relative errors for each indicator.

**Figure 6:**
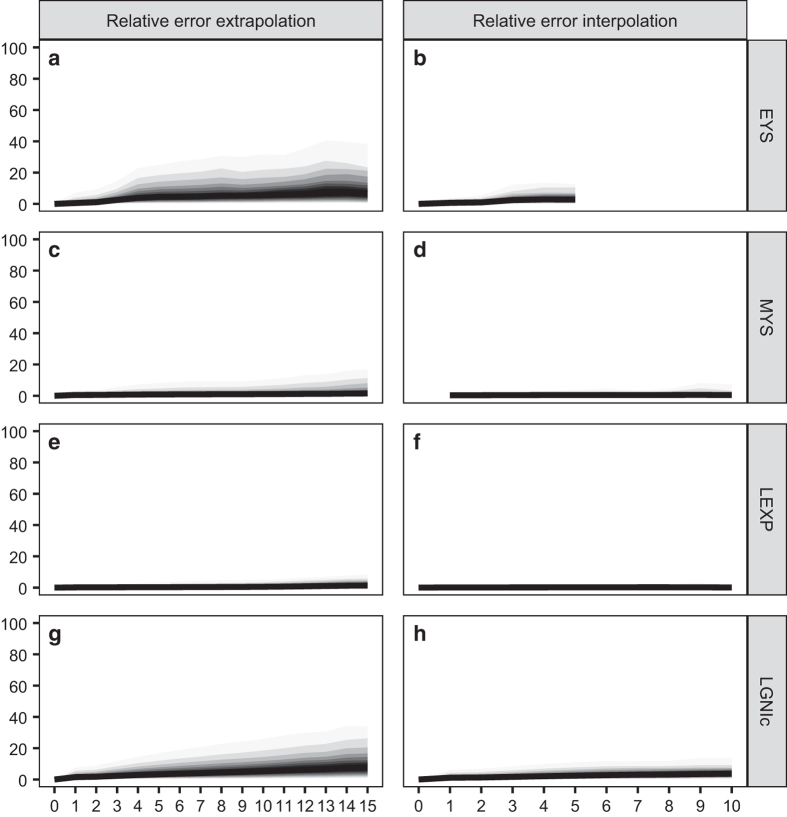
Relative error ventile distributions for the different SHDI indicators. Extrapolation relative errors are shown in the first column and interpolation ones in the second one. The shaded bands indicate the position of the different distribution ventiles. (**a**,**b**) Relative errors for EYS. (**c**,**d**) Relative errors for MYS. (**e**,**f**) Relative errors for LEXP. (**g**,**h**) Relative errors for LGNIc.

Figure 6 shows the different ventiles of those distributions (i.e. the position of centiles 5, 10, …, 90 and 95) as a function of the number of inter- or extrapolation years for the four SHDI indicators separately. The central ventiles (i.e. those indicating the most representative values of the distribution) are coloured in dark grey, and those at the lower and upper extremes are coloured in clearer shades. The first column shows the results for extrapolations up to 15 years (where forward and backward extrapolations have been pooled together) and the second column the results for interpolations up to 10 years (in the last case, 10 years mean that the extreme values that have been used for the interpolation are 20 years apart). Beyond those bounds (and for EYS beyond five years), the number of simulations that the observed data allow is particularly small, thus leading to noisy and unreliable estimates.

As can be seen in Fig. 6, the median of the relative error distribution is remarkably small for all possible extrapolation years for all four indicators. When the number of extrapolation years is at its height (15 years), the medians of the relative error distributions are 6, 2, 1 and 7% for EYS, MYS, LEXP and LGNIc, respectively. For the interpolations, the medians are even smaller. As expected, (a) relative errors tend to be larger in the case of extrapolations, and (b) the longer the inter- or extrapolation period, the larger the relative error tends to be. As indicated by the relative position of the different ventiles, we can see that most relative errors are highly concentrated around the median – except for a relatively small number of cases for long-term EYS and LGNIc extrapolations. For MYS and, specially, LEXP, the range of variability of the relative errors is particularly narrow. If we restrict our attention to short-term extrapolations (i.e. with five or less year steps; see above), the relative errors are within reasonable bounds. Overall, the sizes of the relative errors reported in Fig. 6 tends to be smaller than the ones reported by Kummu *et al.*^7^ in their analogous error analysis.

## Additional information

**How to cite this article**: Smits, J. and Permanyer, I. The Subnational Human Development Database. *Sci. Data*. 6:190038 https://doi.org/10.1038/sdata.2019.38 (2019).

**Publisher’s note**: Springer Nature remains neutral with regard to jurisdictional claims in published maps and institutional affiliations.

## Supplementary Material



## Figures and Tables

**Table 1:** Summary of indicators included in the SHDI.

Dimension	Indicator	Description	Min	Max	Main Sources	Notes
Education	Mean years of schooling of adults aged 25+ (MYS)	Average years of schooling for the population aged 25 or more years.	0	15	Eurostat, GDL-AD, UNDP, …Varying sources (see details in Supplementary information)	When only educational attainment data was available, we imputed the corresponding years of schooling.
Education	Expected years of schooling (EYS)	Number of years of schooling a child of school entrance age can expect to receive, if prevailing patterns of age-specific enrolment rates persist throughout the child’s schooling life	0	18	Eurostat, GDL-AD, UNDP, …Varying sources (see details in Supplementary information)	Lacking for HICs outside EU.
Health	Life expectancy at birth (LIFEX)	Number of years newborn children would live if subject to the mortality risks prevailing for the cross-section of population at the time of their birth.	20	85	Eurostat, GDL-AD, UNDP, …Varying sources (see details in Supplementary information)	In case of missing data, LIFEX was estimated using information on child mortality (see methods section).
Standard of living	(Log of) Gross national income per capita (LGNIc)	(Log of) Sum of value added by all resident producers plus any product taxes (less subsidies) not included in the valuation of output plus net receipts of primary *income* (compensation of employees and property *income*) from abroad	100	75000	Eurostat, GDL-AD, UNDP, …Varying sources (see details in Supplementary information)	LGNIc is based on Purchasing Power Parity (PPP) in 2011 US$. In case of missing data, LGNIc was estimated on the basis of IWI scores (see methods section).

**Table 2:** Record description of the SHDI-Database file.

Record	Description
iso_code	ISO country code 3 digit
country	Country name
year	Year
gdlcode	Region code Global Data Lab
level	Aggregation level (national or subnational)
region	Name of subnational region
shdi	Subnational Human Development Index
healthindex	Health index
incindex	Income index
edindex	Educational index
lifexp	Life expectancy at birth
lgnic	Log Gross National Income per capita PPP in constant 2011 international US$
esch	Expected years schooling of children aged 6
msch	Mean years schooling of population aged 25 and over

**Table 3:** Record description of the SHDI-Starting-Data file.

Record	Description
iso_code	ISO country code 3 digit
country	Country name
year	Year
gdlcode	Region code Global Data Lab
level	Aggregation level (national or subnational)
region	Name of subnational region
source1	Primary data source
source2	Additional data source
lifexp	Life expectancy at birth
lgnic	Log Gross National Income per capita PPP in constant 2011 international US$
esch	Expected years schooling of children aged 6
msch	Mean years schooling of population aged 25 and over

**Table 4:** Record description of the SHDI-Data-Quality file.

iso_code	ISO country code 3 digit
country	Country name
year	Year
gdlcode	Region code Global Data Lab
level	Aggregation level (national or subnational)
region	Name of subnational region
year	Year
miss	Combination of missings
lgnic_polated	LGNIc inter or extrapolated
	*0 Real value*
	*1 Interpolated*
	*2 Extrapolated with past value*
	*3 Extrapolated with future value*
	*4 National value used*
lgnic_polyears	Years over which LGNIc is inter or extrapolated
lifexp_polated	Life expectancy inter or extrapolated
	*0 Real value*
	*1 Interpolated*
	*2 Extrapolated with past value*
	*3 Extrapolated with future value*
	*4 National value used*
lifexp_polyears	Years over which life expectancy is inter or extrapolated
esch_polated	Expected years of schooling inter or extrapolated
	*0 Real value*
	*1 Interpolated*
	*2 Extrapolated with past value*
	*3 Extrapolated with future value*
	*4 National value used*
esch_polyears	Years over which expected schooling is inter or extrapolated
msch_polated	Mean years of schooling inter or extrapolated
	*0 Real value*
	*1 Interpolated*
	*2 Extrapolated with past value*
	*3 Extrapolated with future value*
	*4 National value used*
msch_polyears	Years over which mean years of schooling is inter or extrapolated

**Table 5:** Record description of the UNDP-HDI-Data file.

Record	Description
iso_code	ISO country code 3 digit
year	Year
hdiun	Human Development Index (national)
lifexpun	Life expectancy at birth (national)
gnicun	Gross National Income per capita PPP in 2011 international US$ (national)
expschun	Expected years schooling of children aged 6 (national)
yrschun	Mean years schooling of population aged 25 and over (national)

**Table 6:** Percentage of country-year SHDI estimates depending on the number of dimensions (out of three) in which the corresponding indicators are ‘high-quality’ (i.e. either real or interpolated data).

	All	1990–1999	2000–2017
At least one high-quality value	52.3	34.9	61.9
At least two high-quality values	44.7	19.5	58.5
Based on three high-quality values	34.5	14.6	45.4
